# History of stroke as a predictor of high left atrial fibrosis in patients with persistent atrial fibrillation—insight from the DECAAF II randomized trial

**DOI:** 10.1007/s10840-024-01837-4

**Published:** 2024-07-18

**Authors:** Abdel Hadi El Hajjar, Lilas Dagher, Hadi Younes, Mario Mekhael, Charbel Noujaim, Nour Chouman, Tom Greene, Amitabh C. Pandey, Chao Huang, Nassir Marrouche

**Affiliations:** 1 Tulane Research and Innovation for Arrhythmia Discoveries-TRIAD Center, Tulane University School of Medicine, New Orleans, LA USA; 2 Cleveland Clinic Foundation, Cleveland, OH USA; 3 Emory University School of Medicine, Atlanta, GA USA

**Keywords:** Atrial fibrillation, Stroke, Atrial fibrosis, Stroke predictors

## Abstract

**Background:**

There is a strong relationship between left atrial (LA) remodeling and ischemic stroke (IS) risk in atrial fibrillation (AF) patients. The Efficacy of Delayed Enhancement MRI-Guided Ablation vs. Conventional Catheter Ablation of Atrial Fibrillation (DECAAF-II) is the biggest MRI-based, randomized, multicenter clinical trial performed on persistent AF patients. The aim of this study is to evaluate the relationship between history of stroke and atrial fibrosis in the DECAAF II population.

**Methods:**

Persistent AF patients who underwent Late Gadolinium Enhancement Magnetic Resonance Imaging (LGE-MRI) were included in the study and divided into two different groups: those with a history of stroke and those without. Propensity score matching was performed to adjust for covariates. Atrial fibrosis was compared in both groups. Then, patients were divided into different fibrosis groups, using three different cut-offs of baseline atrial fibrosis: ≥ 15%, ≥ 20%, and ≥ 25%. Univariate logistic regression and adjusted multivariate analysis were performed to assess the effect of clinical characteristics and risk factors on baseline fibrosis.

**Results:**

Eight-hundred forty-three patients were recruited in DECAAF II, of whom 70 (8.3%) had a history of stroke. Patients with history of stroke had a higher prevalence of hypertension (*p* = 0.043), diabetes (*p* = 0.014), and hyperlipidemia (*p* = 0.001). Seventy patients with no history of strokes were matched with patients with history of stroke to adjust for covariates using propensity score analysis. Patients in the stroke group had a significantly higher level of fibrosis than those without (20.2% vs. 8.1%, *p* = 0.017). Increased age was a significant predictor of all three baseline fibrosis classes (≥ 15%, ≥ 20%, and ≥ 25%). Additionally, history of stroke was found to be a predictor of baseline fibrosis ≥ 25% even after adjusting for other clinical characteristics and risk factors (OR = 1.98 [1.14–3.43], *p* = 0.01).

**Conclusions:**

Left atrial fibrosis level greater than 25% correlates with the history of previous stroke episodes in patients with persistent atrial fibrillation.

**Graphical Abstract:**

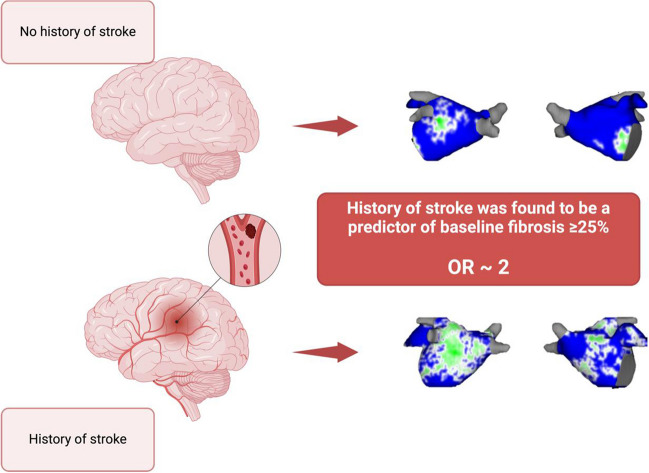

## Introduction

Atrial fibrillation (AF) is the most common cardiac arrhythmia and is associated with increased all-cause mortality and morbidity. Among the multitude of complications associated with AF, embolic strokes remain an important cause of death [1]. In fact, AF patients have five times higher risk of embolic strokes than the general population, and embolic strokes in AF patients are associated with worse outcomes than embolic strokes from other sources.

Clinical risk assessment scores, such as the CHA_2_DS_2_-VASc score, are used to assess stroke risk and select AF patients who might benefit from anticoagulation therapy. However, the CHA_2_DS_2_-VASc score shows variable accuracy in prediction of stroke risk, and its role in clinical decision-making is increasingly being challenged [2]. On the other hand, atrial fibrosis as measured by late gadolinium enhancement magnetic resonance imaging (LGE-MRI) is a quantitative and objective way to assess for atrial disease and has been associated with a higher risk of both catheter ablation (CA) complications [3] and strokes [4–6]. In fact, fibrosis detected on LGE-MRI is predictive of ischemic stroke independent from AF status [7]. However, data regarding the correlation between stroke and atrial fibrosis levels in persistent AF patients remains sparse.

The Efficacy of LGE-MRI-Guided Fibrosis Ablation Versus Conventional Catheter Ablation of Atrial Fibrillation trial (DECAAF II) is the largest to collect LGE-MRI in persistent AF patients prior to catheter ablation [8]. A large subset of the DECAAF II population (*n* = 70) have a history of stroke. We sought to utilize this dataset in studying the correlation between the history of stroke and underlying atrial fibrosis.

## Methods

### DECAAF II protocol

DECAAF II was a multinational, prospective, and randomized trial that encompassed 44 centers across the United States, Europe, and Australia. Between July 2016 and January 2020, a total of 843 patients diagnosed with symptomatic or asymptomatic persistent atrial fibrillation (AF) and undergoing their initial catheter ablation (CA) procedure were enrolled in the study. Follow-up was conducted until February 2021. The patients were randomly assigned to one of two treatment groups: pulmonary vein isolation (PVI) alone, consisting of 422 patients, or PVI combined with MRI-guided atrial fibrosis ablation, involving 421 patients. Patients underwent LGE-MRI before and 3 months following ablation. Details of the study design have been previously described [8]. The primary endpoint of DECAAF II was the first confirmed recurrence of atrial arrhythmia (including atrial fibrillation, atrial flutter, or atrial tachycardia) lasting for at least 30 s after the 90-day blanking period, demonstrated by at least two consecutive 1-lead smartphone ECG device tracings, one positive reading on a clinical 12-lead ECG tracing or ambulatory monitor, or if the patient underwent repeat ablation.

### Late gadolinium enhancement MRI

Patients underwent LGE-MRI within a 30-day window prior to the catheter ablation (CA) procedure. The primary objective of this imaging was to assess the baseline level of fibrosis, which subsequently served as a crucial reference for guiding the ablation procedure in the fibrosis-guided ablation group. Patients underwent a second LGE-MRI 3 months following catheter ablation, aiming to assess ablation-induced scar. The details of the atrial imaging protocol were previously described [3, 9]. MARREK Inc. assisted with image segmentation, processing, and quantification of left atrial fibrosis and subsequent scar formation in the post-ablation scan.

### Design of this sub-analysis

The DECAAF II subpopulation was divided into two distinct groups: patients with a history of stroke (referred to as the “stroke group”) and patients without a history of stroke (referred to as the “control group”). Baseline demographics and co-morbidities were compared between these two groups. The primary focus of the DECAAF II study was to evaluate arrhythmia recurrence, which served as the primary outcome measure for both the stroke and control groups.

Within this sub-analysis, the primary outcome of interest is the assessment of baseline fibrosis. To accomplish this, various parameters were considered, including mean fibrosis, fibrosis stage, and a dichotomous variable indicating low and high fibrosis. Low fibrosis was defined as atrial fibrosis of 25% or less, while high fibrosis was defined as atrial fibrosis exceeding 25%.

### Statistical analysis

All statistical analyses were performed using the Stastical Package for Social Sciences (SPSS) version 27.0.1. Continuous variables were reported as means with standard deviations. Patient characteristics and risk factors such as gender, AF type, and hypertension were summarized as frequencies and percentages. To compare means of continuous variables, *t*-test or Mann Whitney test were performed depending on normality of the distribution. To compare percentages of categorical variables, Pearson chi square test was performed. In our study, we conducted propensity score matching to reduce confounding biases in the two initial groups, which exhibited significant differences in several co-morbidities—namely, hypertension, diabetes, and hyperlipidemia. Propensity score was applied by first calculating the propensity scores for each individual in both cohorts. These scores were derived based on the likelihood of having each of the specified conditions, using logistic regression models. Subsequently, individuals from each group were matched based on their scores, ensuring that each matched pair had similar values for these comorbidities. The propensity scores matching method used is nearest neighbor matching, which pairs each treated subject with a control subject who has the closest propensity score. Survival analysis (using log-rank analysis/Kaplan-Meir curves) was performed to determine the original DECAAF II primary endpoint in both groups. Finally, multiple logistic regression was performed that relate the dichotomous outcomes based on the different fibrosis % cutoffs to the predictor. To do so, all DECAAF II patients were included in the analysis and divided into three different groups based on baseline left atrial fibrosis levels (≥ 15%, ≥ 20%, and ≥ 25%). Univariate logistic regression and adjusted multivariate analysis were performed to assess the effects of clinical characteristics and risk factors on baseline fibrosis. In other terms, we used a forward stepwise model: unadjusted (model 1); an adjusted model for age, sex, CAD, CHF, hypertension, and diabetes, based on atrial fibrosis ≥ 15%; an adjusted model for age, sex, CAD, CHF, hypertension, and diabetes, based on atrial fibrosis ≥ 20%; and an adjusted model for age, sex, CAD, CHF, hypertension, and diabetes, based on atrial fibrosis ≥ 25%.

**Fig. 1:**
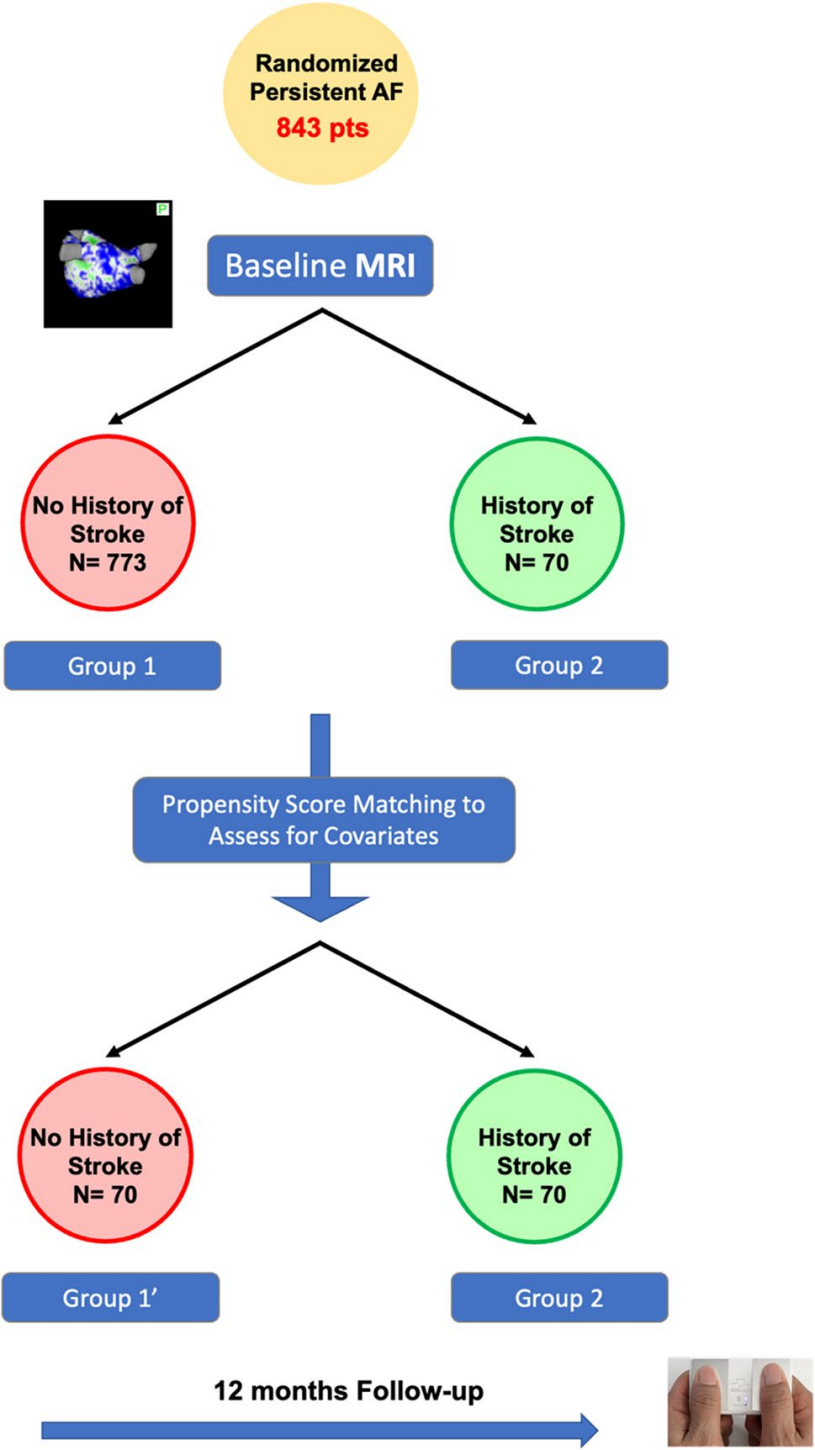
Study design

The study methodology is summarized in Fig. 1.

## Results

### DECAAF II population demographics

Patients in the DECAAF II trial all had persistent AF and were on average 62.1 years old. One-hundred-seventy-eight patients (21.1%) were females. Forty-seven percent of patients were on antiarrhythmic drugs prior to ablation, 74.1% were on beta blockers, and 96.0% were anticoagulated. The DECAAF II population had the following co-morbidities (proportion): hypertension (58.6%), tobacco smokers (36.9%), hyperlipidemia (34.2%), congestive heart failure (19.1%), coronary artery disease (12.7%), diabetes mellitus (10.1%), vascular disease (10.0%), coronary artery bypass graft (1.5%), and rheumatic fever (1.3%). Around 83.7% of the patients were previously cardioverted, and 58.1% had previously failed anti-arrhythmic treatment.

### Baseline demographics of the two groups

A total of 843 patients were included in the study; 70 of whom had a history of stroke (stroke group) and 773 did not (control group). Patients with history of stroke were significantly older than those without (65.6 ± 7.3 vs. 61.8 ± 9.2 years, *p* < 0.001). Patients with a history of stroke had higher prevalence of hypertension (70.0% vs. 57.6%, *p* = 0.043), diabetes mellitus (18.6% vs. 9.3%, *p* = 0.014), and hyperlipidemia (52.4% vs. 32.6%, *p* = 0.001). Gender and treatment randomization were equally distributed between groups (*p* = 0.325 and *p* = 0.928, respectively). There was no significant difference in other comorbidities between the two groups (*p* > 0.05). All baseline characteristics are further described in Table 1.

**Table 1:** Baseline demographics in patients without a history of stroke (control group) and patients with history of stroke (stroke group)

	Control group	Stroke group	*p* value
Age (mean ± SD)	61.8 ± 9.2	65.6 ± 7.3	**<0.001**
Female sex	20.7%	25.7%	0.325
Treatment strategy	51.0%	48.6%	0.928
Anti-arrhythmic meds	47.1%	45.7%	0.825
Anticoagulation	95.7%	98.6%	0.247
Hypertension	57.6%	70%	**0.043**
Congestive heart failure	18.9%	21.4%	0.605
Diabetes mellitus	9.3%	18.6%	**0.014**
Vascular disease	10.0%	10.0%	0.992
Tobacco	37.1%	34.3%	0.637
Coronary artery disease	12.0%	20.0%	0.055
Coronary artery bypass graft	1.6%	1.4%	0.936
Mitral valve disease	5.7%	8.6%	0.329
Hyperlipidemia	32.6%	51.4%	**0.001**
Rheumatic fever	1.2%	2.9%	0.232
Previously cardioverted	83.8%	82.9%	0.833
Failed antiarrhythmics	57.6%	64.3%	0.275

### Left atrial fibrosis proportion in both groups

- a. Mean fibrosis

**Fig. 2:**
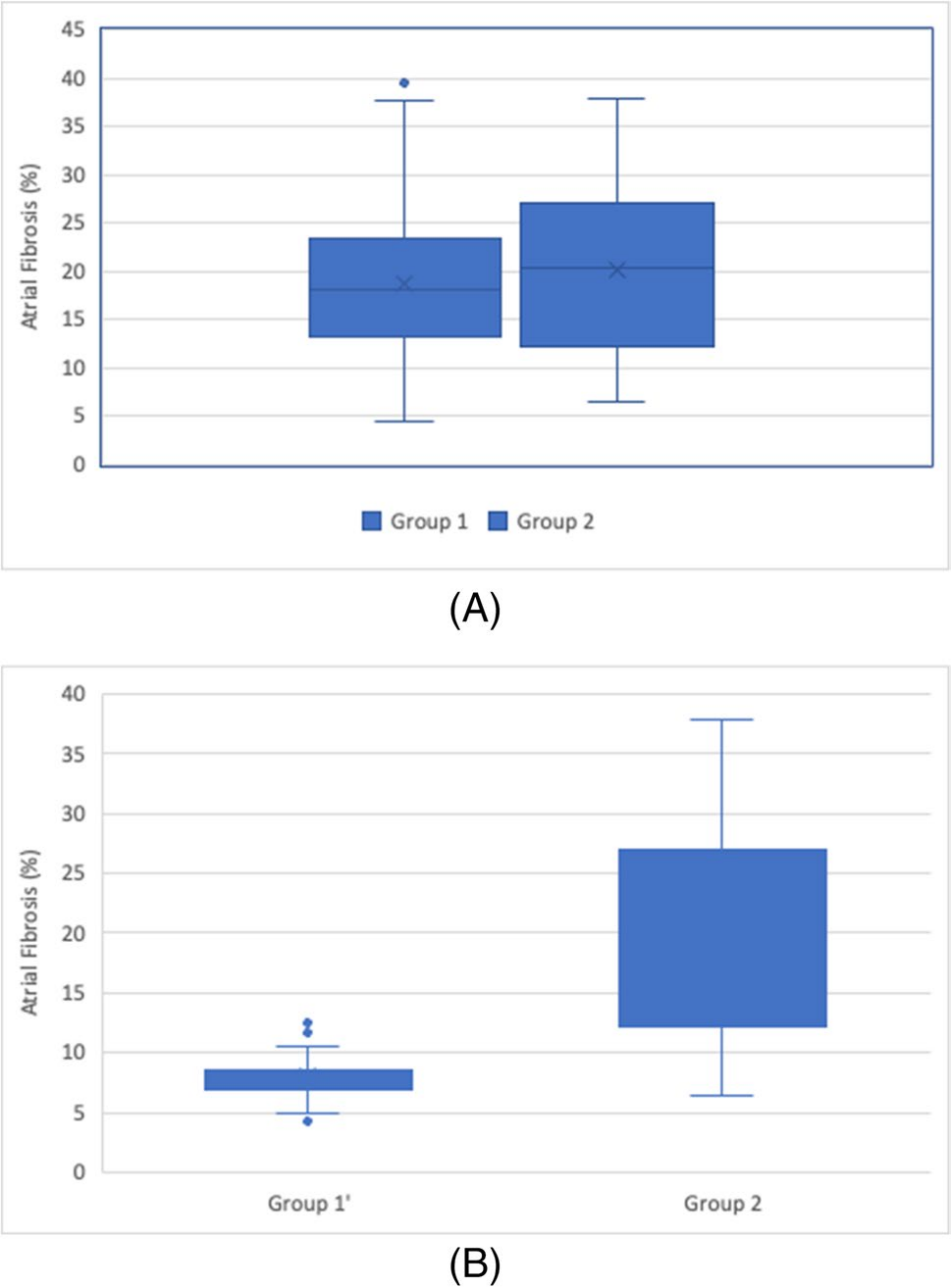
Baseline fibrosis distribution in patients without and with history of stroke (groups 1 and 2, respectively) in the study population before (**A**) and after propensity matching (**B**)

At baseline, patients with history of stroke had significantly higher levels of fibrosis than those without (20.2% vs. 18.7%, *p* = 0.017). Fibrosis proportions are shown in Fig. 2A.

- b. Fibrosis stage

There was a significant difference in proportion between the two groups across fibrosis stages (*p* = 0.033). In the control group, 11.6% were stage I, 47.6% were stage II, 33.2% were stage III, and 7.5% were stage IV. On the other hand, in the stroke group, 11.4% were stage I, 37.1% were stage II, 34.3% were stage III, and 17.1% were stage IV.

- c. Low and high fibrosis groups

There were significantly more patients with high fibrosis (> 25%), the stroke group than in the control group (31.4% vs. 17.9%, *p* = 0.006).

### Fibrosis proportion after propensity score matching

To correct for potential confounding factors and ensure a balanced comparison between our study groups, propensity score matching was performed. Specifically, 70 patients with no history of stroke were matched with 70 patients who had a history of stroke.

- a. Baseline demographics in the matched groups

All baseline characteristics in the matched groups are shown in Table 2.

- b. Mean Fibrosis in the matched groups

**Table 2:** Baseline demographics, after propensity score matching, in patients without history of stroke (propensity matched control group) and patients with history of stroke (stroke group)

	Propensity-matched control group	Stroke group	*p* value
Age (mean ± SD)	58.8 ± 9.6	65.6 ± 7.3	0.053
Female sex	13.0%	25.7%	0.054
Treatment strategy	51.4%	48.6%	0.482
Anti-arrhythmic meds	52.9%	45.7%	0.398
Anticoagulation	97.1%	98.6%	0.559
Hypertension	70.0%	70.0%	1.00
Congestive heart failure	12.4%	21.4%	0.178
Diabetes mellitus	18.6%	18.6%	1.00
Vascular disease	7.1%	10.0%	0.546
Tobacco	47.1%	34.3%	0.122
Coronary artery disease	8.6%	20.0%	0.053
Coronary artery bypass graft	2.9%	1.4%	0.559
Mitral valve disease	5.7%	8.6%	0.512
Hyperlipidemia	40.0%	51.4%	0.175
Rheumatic fever	0.0%	2.9%	0.154
Previously cardioverted	82.9%	82.9%	1.00
Failed antiarrhythmics	71.4%	64.3%	0.366

Patients with history of stroke in the matched group had a significantly higher level of fibrosis than those without history of stroke (20.2% vs. 8.1%, *p* = 0.017). Fibrosis proportion is shown in Fig. 2B.

- c. Fibrosis stages in the matched groups

There was a significant difference in the proportion of Utah stages of fibrosis between the two groups (*p* < 0.001). In patients with no history of stroke after propensity score matching, 88.6% were stage I, 11.4% were stage II, 0.0% were stage III, and 0.0% were stage IV. On the other hand, in patients with a history of stroke, 11.4% were stage I, 37.1% were stage II, 34.3% were stage III, and 17.1% were stage IV.

- d. Low and high fibrosis in the matched groups

**Fig. 3:**
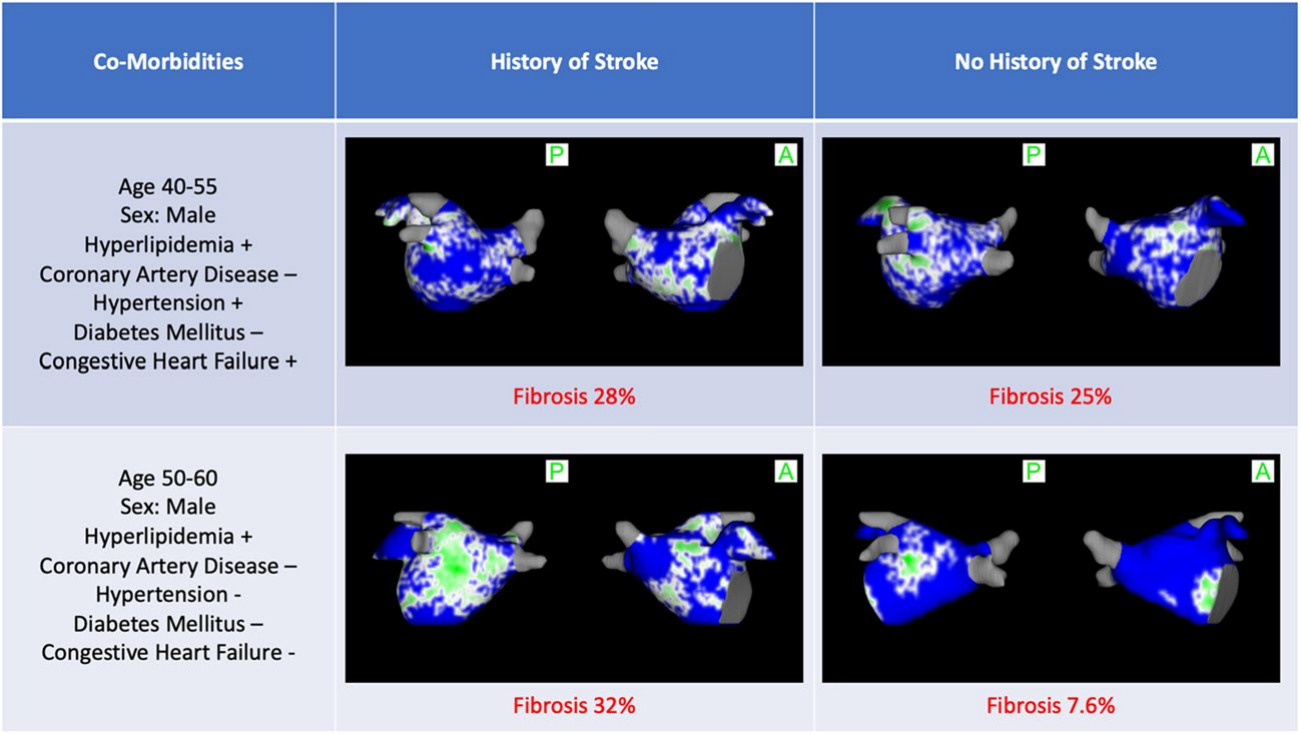
Baseline LGE-MRI scans of two patients with a history of stroke and two patients without a history of stroke with similar comorbidities

There were significantly more patients with high fibrosis (> 25%) in the stroke group than in matched control group (31.4% vs. 0.0%, *p* < 0.001). Baseline LGE-MRI scans of two patients with a history of stroke and two patients without a history of stroke with similar comorbidities are shown in Fig. 3.

### Atrial fibrillation recurrence in both groups

**Fig. 4:**
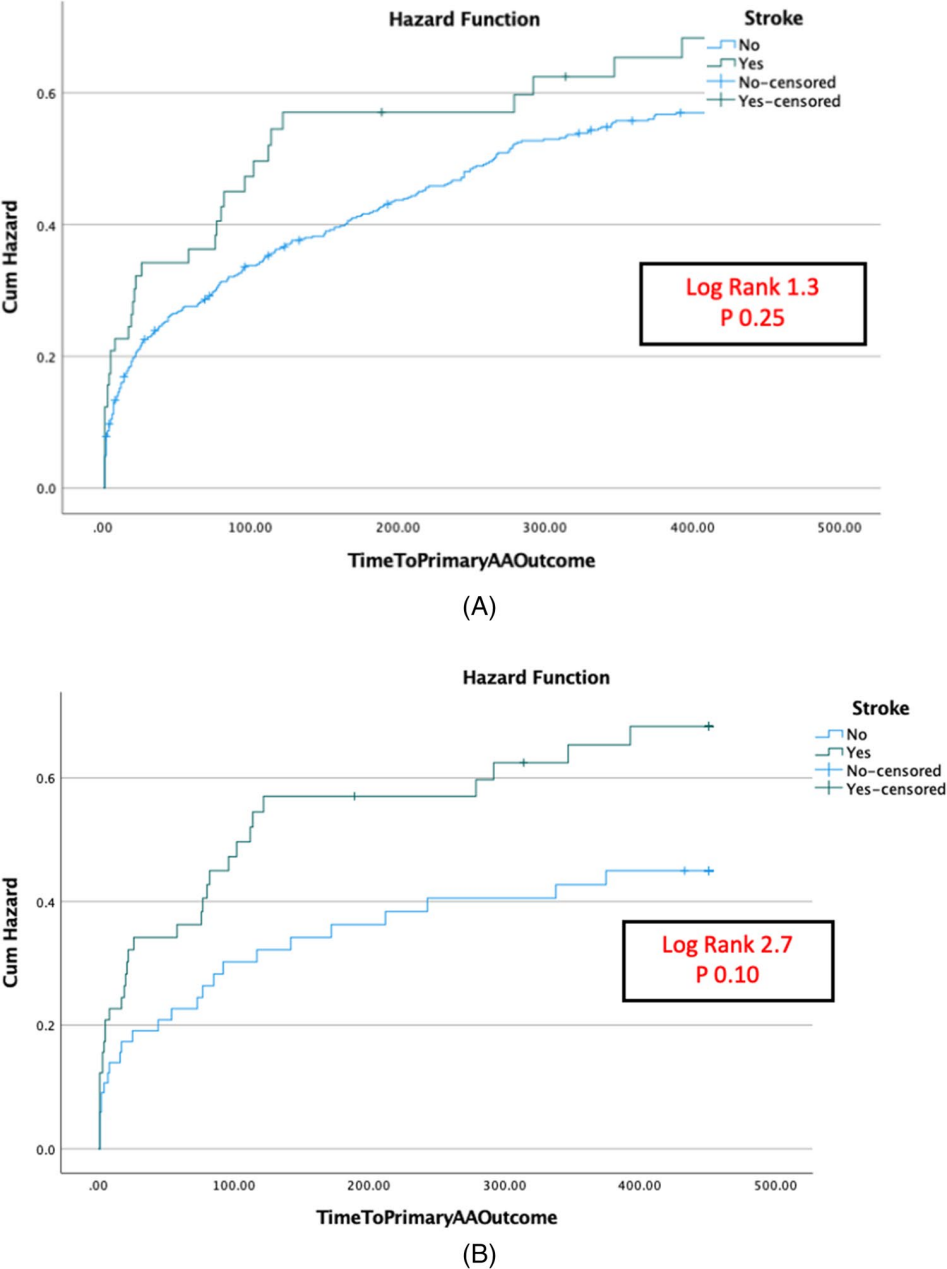
Kaplan-Meir curves showing atrial arrhythmia recurrence with and without a history of stroke in the study population before (**A**) and after propensity matching (**B**)

Patients with history of stroke tended to have a higher atrial arrhythmia recurrence than patients without a history of stroke (log rank 1.3, *p* = 0.25) (Fig. 4A). After propensity score matching, the stroke group also tended to have a higher atrial arrhythmia recurrence than patients without a history of stroke (log rank 2.7, *p* = 0.1) (Fig. 4B).

### Predictors of atrial fibrosis in DECAAF II

Univariate logistic regression and adjusted multivariate analysis were performed to assess the effects of clinical characteristics and risk factors on baseline fibrosis in three different groups based on fibrosis levels (≥ 15%, ≥ 20%, and ≥ 25%). Increased age was a significant predictor for all three baseline fibrosis classes: odds ratio (OR) = 1.03 (1.01–1.05), *p* < 0.001 for fibrosis levels ≥ 15%, OR = 1.03 (1.01–1.05) *p* = 0.001 for fibrosis levels ≥ 20%, and OR = 1.04 (1.02–1.06), *p* < 0.001 for fibrosis levels ≥ 25. Additionally, history of stroke was found to be a predictor for baseline fibrosis ≥ 25%, even after adjusting for clinical characteristics and risk factors (OR = 1.98 (1.14–3.43), *p* = 0.014). Multivariate analyses for all three cut-offs are shown in Table 3.

**Table 3:** Logistic regression results of stroke’s effect on baseline fibrosis cutoff points

Dependent variable	Effect	Unadjusted odds ratio (CI)	*p* value	Adjusted odds ratio (CI)	*p* value
Baseline fibrosis ≥ 15	Stroke	1.03 (0.61, 1.74)	0.9011	0.95 (0.56, 1.62)	0.8637
	Age (years)			1.03 (1.01, 1.05)	0.0004
	Male sex			1.28 (0.88, 1.85)	0.1904
	CAD			0.73 (0.47, 1.14)	0.1624
	CHF			0.9 (0.63, 1.3)	0.5792
	HTN			1.1 (0.81, 1.48)	0.5485
	DM			0.87 (0.54, 1.41)	0.5764
Baseline fibrosis ≥ 20	Stroke	1.54 (0.94, 2.52)	0.0838	1.43 (0.87, 2.35)	0.1616
	Age (years)			1.03 (1.01, 1.05)	0.0011
	Male			1.25 (0.87, 1.78)	0.2249
	CAD			0.76 (0.49, 1.17)	0.2131
	CHF			1.02 (0.72, 1.46)	0.9025
	HTN			1.13 (0.84, 1.51)	0.4180
	DM			0.95 (0.6, 1.51)	0.8373
Baseline fibrosis ≥ 25	Stroke	2.12 (1.24, 3.63)	0.0061	1.98 (1.14, 3.43)	0.0146
	Age (years)			1.04 (1.02, 1.06)	0.0004
	Male			1.33 (0.85, 2.08)	0.2148
	CAD			0.74 (0.43, 1.29)	0.2936
	CHF			0.81 (0.51, 1.29)	0.3780
	HTN			1.05 (0.72, 1.51)	0.8112
	DM			0.82 (0.45, 1.49)	0.5099

*CAD*, coronary artery disease; *CHF*, congestive heart failure; *HTN*, hypertension; *DM*, diabetes mellitus

## Discussion

In the present study, we describe two major findings. First, we found a correlation between history of stroke and atrial fibrosis in patients with persistent AF. This association persisted even after adjusting for multiple covariates. More specifically, in our cohort, patients with atrial fibrosis ≥ 25% were two times more likely to have a history of stroke than those with atrial fibrosis < 25%. Second, we identified age as a significant predictor of increased risk of atrial fibrosis in patients with persistent AF.

### The association between atrial fibrosis and ischemic stroke in patients with atrial fibrillation

In our analysis, patients with history of stroke had significantly greater levels of fibrosis than those without. Pathological structural and functional alterations of the LA and LAA associated with underlying LA fibrosis have been studied as predictors and risk factors for cardioembolic strokes, independent of arrhythmic status. Multiple imaging techniques were used to study LA pathology markers such as cardiac magnetic resonance imaging (CMR), echocardiography, and computed tomography (CT). LA pathology markers have been shown to improve the predictive value of traditional risk assessment scores [10–12]. When adding atrial fibrosis to stroke risk stratification, the area under the curve (AUC) for CHADS2 and CHA2DS2-VASc scores shifted from 0.58 to 0.77 and 0.65 to 0.78, respectively [4].

Previous work by Akoum et al. showed that atrial fibrosis is correlated with an increased risk of thrombus in the left atrial appendage [5]. In addition, King et al. demonstrated that increased atrial fibrosis was associated with increased risk of major adverse cardiovascular and cerebrovascular events (MACCE) [6]. Daccarett et al. showed similar findings on a cohort of 387 patients [4] and found that patients with history of stroke have 24.4% atrial fibrosis on average [13]. Similarly, our findings confirm these previous results, as patients with history of stroke had a mean atrial fibrosis of 20.2%. Our study stands out in several significant ways. Firstly, our cohort specifically focuses on persistent atrial fibrillation (AF) patients, providing valuable insights into this specific population. Additionally, our study holds the distinction of being the first to examine various cut-off points of atrial fibrosis and its correlation with stroke history. This pioneering approach adds a new dimension to the existing literature and offers a deeper understanding of the relationship between atrial fibrosis and stroke.

LA functional analysis is an area of study that holds significant prognostic value. Previous research conducted by Inoue et al. has demonstrated a clear association between decreased atrial strain and an increased risk of stroke in patients with atrial fibrillation (AF). This finding highlights the importance of assessing atrial strain as a potential marker for identifying individuals at higher risk of stroke. [14]. Leong et al. showed that a decrease of LA emptying fraction by 1%, as determined by speckle-tracking echocardiography, can increase the risk of stroke by 7% [15]. Moreover, Habibi et al. showed that an LA emptying fraction inferior to 40% is associated with increased risk of stroke in AF patients [16].

Similarly, morphology of the LA correlates with stroke history. Bisbal et al. showed that patients with a history of stroke tend to have a more spherical shape of the LA than patients with no previous history of stroke [17]. In that study, they showed that adding atrial shape to the CHA2DS2-VASc score can increase the area under the curve (AUC) from 0.59 to 0.67. Tan et al. also showed a positive correlation between atrial sphericity, as estimated using transthoracic echocardiogram, and stroke risk [18]. Consequently, Cates et al. established shape classification of the LA, with shape class 3 and 4 being associated with increased stroke risk [19, 20].

Another interesting parameter that could enhance stroke prediction models in AF is AF burden. Studies such as TRENDS and ASSERT showed that higher AF burden correlates with an increased risk of stroke [21, 22]. In addition, LA fibrosis has been associated with both increased AF recurrence and AF burden following ablation [3, 23]. Future studies should explore whether AF burden can serve as an indicator of LA fibrosis and whether it can be integrated into stroke prediction models for patients with AF.

To sum up, previous studies and the presented DECAAF II data highlight the strong correlation between atrial myopathy and stroke risk. Atrial myopathy is an evolving concept that is best defined as an anatomical and structural remodeling of the left atrium, with subsequent LA function impairment, creating a substantial substrate for arrhythmogenesis [24]. To our knowledge, this is the first study to establish a cut-off point as a history of stroke was found to be predictor for baseline fibrosis ≥ 25% even after adjusting for other clinical characteristics and risk factors (OR = 1.98 (1.14–3.43), *p* = 0.014).

Our findings are of high clinical relevance as current stroke risk assessment scores in AF population can underperform in certain patient populations [25]. Conflicting data report a high stroke risk in AF patients even with low CHA2DS2VASc score, ranging from 0.6 to > 2.0% [26–28]. A recent systemic review showed an annual ischemic stroke rate of 0.68% in patients with CHA2DS2VASc score of 0 and 1.61% in patients with CHA2DS2VASc score of 1 [27]. This discrepancy between the predicted and actual stroke risks leaves many patients mistakenly deemed low risk without prophylactic anti-coagulation, with deleterious impact on their health. Moreover, current cardioembolic stroke risk assessment scores are based on non-specific clinical and demographic criteria, thus showing predictive value for ischemic stroke even in non-AF populations [29, 30]. Overall, there is a significant demand for more precise estimation of cardioembolic stroke risk in order to accurately assess the likelihood of events and enhance assessment scores. We suggest integrating high atrial fibrosis (> 25%) into stroke risk predictions for patients diagnosed with atrial fibrillation (AF).

### Atrial arrhythmia recurrence in patients with history of stroke

To our knowledge, this is the first study to hint at the relationship between history of stroke and catheter ablation outcomes. In our cohort, after propensity score matching, patients with a history of stroke had a trend towards more arrhythmia recurrence than controls but that did not reach significance. A larger group of patients may yield significant results. Those results might be driven by the fact that patients with previous history of stroke had significantly higher levels of baseline atrial fibrosis than controls. Previous studies, such as the DECAAF I trial, have already established high baseline atrial fibrosis as a predictor of arrhythmia recurrence after catheter ablation [3].

### Limitations

Our study has several limitations. First, we studied the relationship between history of stroke prior to LGE-MRI and atrial fibrosis. This can establish the strong correlation between ischemic stroke and high atrial fibrosis (> 25%), but it cannot confirm causality. Future studies should assess the temporality of this relationship in order to understand the interaction between stroke and atrial disease. Second, DECAAF II has relatively short follow-up period (12–18 months) to detect new onset stroke after catheter ablation. Studies with longer follow-up period should be performed to study the effect of extensive LA ablation and subsequent scar formation, on stroke incidence.

## Conclusion

In patients with persistent atrial fibrillation, there is a notable correlation between a history of stroke and a high degree of atrial fibrosis, as determined through LGE-MRI assessment. More specifically, individuals who have experienced previous stroke episodes face a doubled risk of having an atrial fibrosis level of 25% or greater.

## Data Availability

The data that support the findings of this study are available from the corresponding author, NM, upon reasonable request.
